# Entomological findings in a focus of visceral leishmaniasis transmission in Embu das Artes Municipality: Contributions for prevention and surveillance

**DOI:** 10.1590/0037-8682-0156-2026

**Published:** 2026-09-18

**Authors:** Sarah Kovalenkinas Xavier de Lima, Rodrigo Accioli Almeida, Fredy Galvis Ovallos, Eunice Aparecida Bianchi Galati

**Affiliations:** 1 Universidade de São Paulo, Faculdade de Saúde Pública, Programa de Pós-graduação em Entomologia em Saúde Pública, São Paulo, SP, Brasil.; 2 Universidade Estadual do Centro-Oeste, Departamento de Geografia, Guarapuava, PR, Brasil.; 3 Universidade de São Paulo, Faculdade de Saúde Pública, Departamento de Epidemiologia, São Paulo, SP, Brasil.

**Keywords:** Phlebotominae, Vectors, Leishmaniasis, Canine visceral leishmaniasis

## Abstract

**Background:**

Leishmaniasis occurs in Embu das Artes, where *Lutzomyia longipalpis* is absent, but secondary vectors are present.

**Methods:**

Sandflies were collected using Shannon and CDC (Center for Disease Control) light traps in a household with seropositive dogs; presence of flagellates was investigated by dissection.

**Results:**

From 11 species, 1,224 sand flies were collected, predominantly *Pintomyia* (*Pi.*) *fischeri* (88.6%) and *Migonemyia* (*Mg*.) *migonei* (8.4%). Shannon traps captured most specimens, mainly in peridomicile. Abundance was higher during spring/summer. Flagellates were detected only in *Evandromyia* (*Ev*.) *edwardsi*, with a 0.13% overall infection rate.

**Conclusion:**

*Pi. fischeri* predominance and kennel association of *Mg. migonei* reinforce their epidemiological importance for local leishmaniasis transmission.

Leishmaniases are a group of infectious diseases caused by several species of the genus*Leishmania*, which are transmitted to vertebrate hosts through the bite of infected sand fly females (Diptera: Pyschodidade). In Brazil, leishmaniases are broadly distributed, with the cutaneous form being more frequent in the North and the visceral form in the Northeast^1^. 

In São Paulo State, leishmaniasis has been reported in more than 150 of its 645 municipalities, including 38 of the 39 municipalities that comprise the São Paulo Metropolitan Region (RMSP), where 916 cases of cutaneous leishmaniasis (CL) were reported between 2007 and 2023, of which 197 were autochthonous and occurred in five municipalities. Meanwhile, 16 municipalities reported visceral leishmaniasis (VL), totaling 321 cases during the same period, seven of which were autochthonous^1^. The municipality of Embu das Artes has been an endemic area for canine VL since the early 2000s. The Municipal Zoonosis Control Center (CCZ) conducts reservoir surveillance, with diagnostic confirmation performed by the Adolfo Lutz Institute. Confirmed canine VL cases ranged from 12 to 115 annually between 2013 and 2022^2^. Furthermore, the principal vector of VL, *Lutzomyia longipalpis*, is absent from the municipality, although it has been reported in three municipalities within the RMSP^3^.

*Pintomyia* (*Pi*.) *fischeri* is the most frequently collected sand fly species in the municipality and is naturally infected with *Leishmania infantum* ^4^. Ecological studies are essential for understanding the transmission dynamics of VL, as well as for identifying and incriminating alternative vector species^5^, thereby providing a scientific basis for entomological surveillance. Therefore, this study aimed to characterize the sand fly fauna associated with a focus on canine visceral leishmaniasis (CVL) in Embu das Artes and to assess evidence supporting the participation of secondary vector species in local parasite transmission.

This municipality is located 30 km west of São Paulo city and borders the municipalities of São Paulo, Cotia, Itapecerica da Serra, and Taboão da Serra (Figure 1A-B ). Although it is predominantly urban, it contains areas that preserve extensive remnants of primary and secondary Atlantic Forest vegetation under a subtropical climate. The study area was selected for entomological investigation following the identification by the CCZ of three autochthonous cases of CVL in three households in the Embu Colonial neighborhood in June 2022. Among these, one household was selected for sand fly collections based on the residents' consent, the presence of environmental conditions favorable for sand flies (a preserved forest area), accessibility to sampling sites, and the presence of dogs. 

**FIGURE 1:**
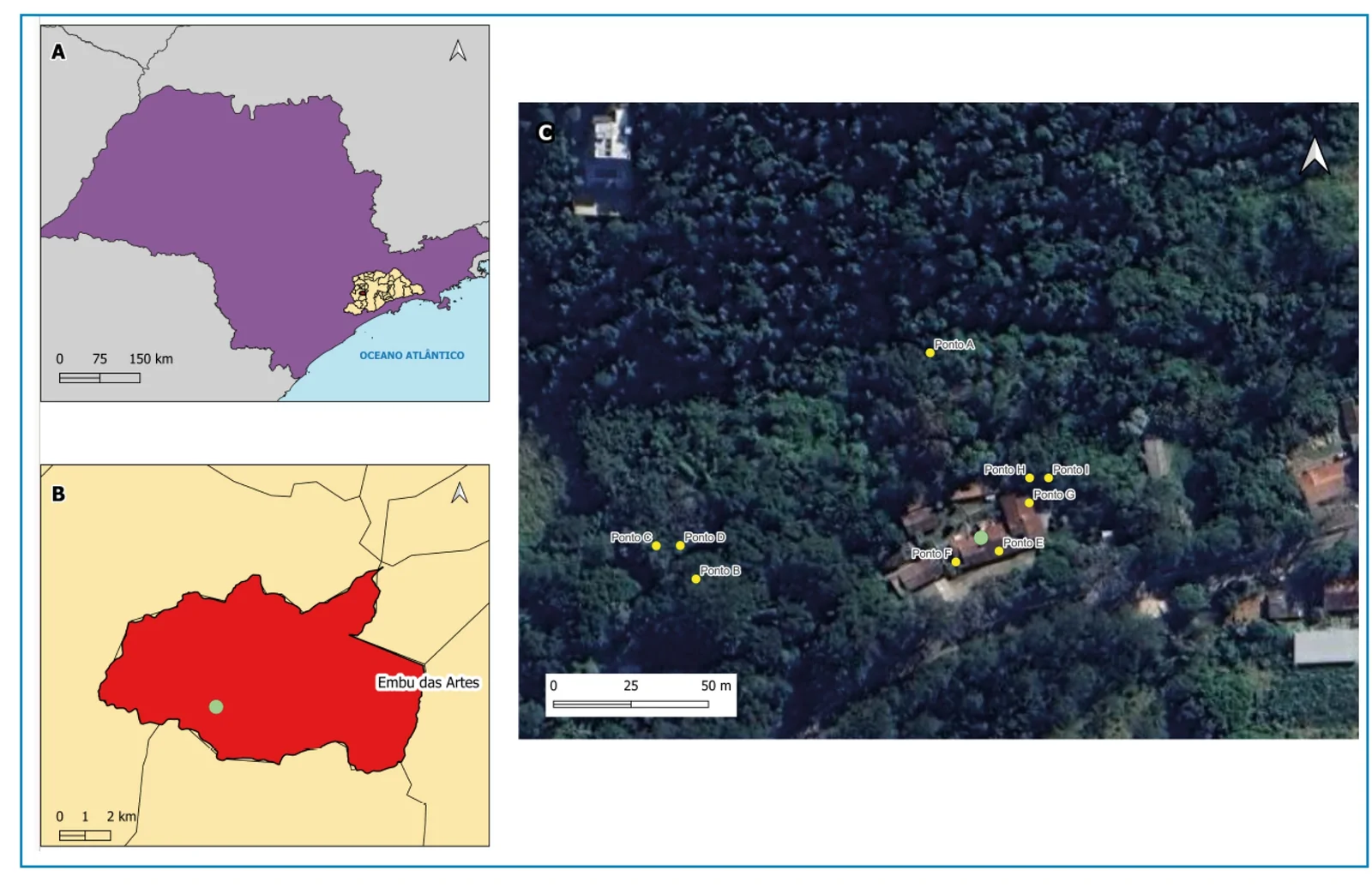
Location of the study area and trap installation points. A: State of São Paulo and RMSP, São Paulo Metropolitan Region. B: Collection location in the municipality of Embu das Artes. C: Trap points.

Capture sites were selected to characterize the sand fly fauna in the forest fragment and to identify the species most closely associated with the intra- and peridomiciliary environments. Sand fly collections were conducted monthly for a single night from July 2022 to December 2023, except in September 2022 and November 2023, using CDC (Center for Disease Control) light traps operated from 6:00 PM to 6:00 AM the following day and Shannon traps operated from 5:00 PM to 9:00 PM. During the first 6 months, collections were conducted at three sites (A, B, and E) monitored with CDC light traps, including two in the forest fragment and one in the peridomicile. Additionally, two sites (C and D) located within the forest fragment were sampled using black and white Shannon traps^6^ (Figure 1C). However, periods of heavy rainfall limited access to this site. Therefore, from January 2023 onward, collections were continued at one remaining site (A) within the forest fragment using a CDC light trap, while four sites near the household (E, F, G, H, and I) were monitored using three CDC light traps (E, F, and G) and two Shannon traps (H and I). The positions of the black and white Shannon traps were alternated monthly to minimize potential bias associated with trap location.

Captured females were anesthetized at low temperature (4°C for 4 min) and dissected, with simultaneous species identification. Undissected specimens were identified using the taxonomic key of Galati^7^. When flagellates were observed, the gut contents were inoculated into Schneider's medium supplemented with 10% heat-inactivated fetal bovine serum (Gibco, USA), 1% L-glutamine, 0.01 µg/mL streptomycin, 10,000 IU/mL penicillin, and 2% sterile human urine. The cultures were then incubated at 25°C and examined daily for 20 days. Additionally, DNA detection was attempted using the extraction protocol described by Galvis-Ovallos et al.^8^ and the PCR conditions described by Guimarães et al.^9^.

To evaluate differences in the attractiveness of black and white Shannon traps for the two most abundant species, the Mann-Whitney test was used. The relationship between sand fly abundance and climatic variables was assessed using Spearman's rank correlation coefficient. Climatic variables included relative humidity on the sampling day, total rainfall, mean temperature during the 15 and 30 days preceding each collection, and mean temperature on the sampling day. Statistical significance was set at p < 0.05. Climatic data were obtained from the Campo Limpo Meteorological Station, operated by the *Centro de Gerenciamento de Emergências Climáticas* of the Municipality of São Paulo.

A total of 1,224 sand flies (353 males and 871 females), representing 11 species distributed across seven genera, were collected. *Pi. fischeri* was the predominant species, accounting for 88.6% of all specimens, followed by *Migonemyia* (*Mg.*) *migonei* (8.4%). The remaining nine species were represented by fewer than 10 specimens each. Regarding temporal distribution, the highest numbers of specimens were collected in August, October, and December 2023, accounting for 50% of all specimens collected. The distribution of specimens by species, sex, and month of collection is presented in Table 1. 

**TABLE 1:** Distribution of sandflies in Embu das Artes from July 2022 to December 2023, according to the month of capture, species, and sex.

Specie	Year	2022						2023												Subtotal	Total	%
	Sex/Month	jul	aug	sep	oct	nov	dec	jan	feb	mar	apr	may	jun	jul	aug	sep	oct	nov	dec			
*Br. pintoi*	M	1	-	...	-	1	-	-	1	-	-	-	-	-	-	-	-	...	-	3	3	0.2
	F	-	-	...	-	-	-	-	-	-	-	-	-	-	-	-	-	...	-	0		
*Ev. edwardsi*	M	-	-	...	-	-	-	-	1	-	-	-	-	-	-	-	-	...	-	1	4	0.3
	F	-	-	...	2	-	-	-	-	-	-	-	1	-	-	-	-	...	-	3		
*Ex. firmatoi*	M	-	-	...	-	-	-	-	-	-	-	-	-	-	-	-	-	...	1	1	8	0.7
	F	-	-	...	-	1	-	-	-	-	1	-	-	-	4	-	1	...	-	7		
*Mg. migonei*	M	-	-	...	-	2	1	7	15	4	12	3	-	-	11	1	13	...	1	70	103	8.4
	F	-	-	...	-	2	2	2	9	1	1	1	-	-	3	2	8	...	2	33		
*Pa. baratai*	M	-	-	...	-	-	-	-	-	-	-	-	-	-	-	-	-	...	-	0	1	0.1
	F	1	-	...	-	-	-	-	-	-	-	-	-	-	-	-	-	...	-	1		
*Pa. lanei*	M	-	-	...	-	-	-	-	-	-	-	-	-	-	-	-	1	...	-	1	2	0.2
	F	-	-	...	-	-	-	-	-	-	-	-	-	-	-	-	1	...	-	1		
*Pa. pascalei*	M	-	-	...	-	-	-	-	-	-	-	-	-	-	-	-	-	...	-	0	2	0.2
	F	-	-	...	-	-	-	-	2	-	-	-	-	-	-	-	-	...	-	2		
*Pi. fischeri*	M	-	31	...	-	4	6	24	4	-	17	-	-	-	44	10	30	...	106	276	1084	88.6
	F	1	23	...	16	22	4	65	47	12	121	4	19	7	132	67	155	...	113	808		
*Pi. monticola*	M	-	-	...	-	-	-	-	-	-	-	-	-	-	-	-	-	...	-	0	8	0.7
	F	-	-	...	-	-	-	-	-	-	-	-	-	-	8	-	-	...	-	8		
*Ps. ayrozai*	M	-	-	...	-	-	-	-	-	-	-	-	-	-	-	-	-	...	1	1	6	0.5
	F	-	-	...	-	-	2	-	-	2	-	-	-	-	-	-	-	...	1	5		
*Ps. lloydi*	M	-	-	...	-	-	-	-	-	-	-	-	-	-	-	-	-	...	-	0	3	0.2
	F	-	-	...	-	-	-	-	1	1	-	-	-	-	-	-	-	...	1	3		
**Total**		**3**	**54**	...	**21**	**32**	**15**	**98**	**80**	**20**	**152**	**8**	**20**	**7**	**202**	**80**	**209**	...	**224**		**1224**	**100**

“...” traps were not installed, M: male, F: female. “-” zero, *Br.: Brumptomyia*, *Ev.: Evandromyia*, *Ex.: Expapillata*, *Mg.: Migonemyia*, *Pa.: Psathyromyia*, *Pi: Pintomyia*, *Ps.:* Psychodopygus.

Regarding the collection method, 80% of all specimens (979) were collected using Shannon traps, of which 72.5% were females. Although the black Shannon trap accounted for 60% (594 specimens) of all specimens collected with this method, no significant difference in attractiveness was observed between the black and white Shannon traps (Mann-Whitney test, U = 144.5, p = 0.58). Among the less abundant species, *Psathyromyia baratai* was collected exclusively in the white Shannon trap, *Expapillata firmatoi* was collected exclusively in the black Shannon trap. Regarding the collection environment, traps located in the peridomicile captured a higher mean number of specimens than did those located in the forest fragment (Table 2).

**TABLE 2:** Frequency (number and percentage) and average (number/trap/night) of specimens of both sexes of *Pintomyia fischeri*, *Migonemyia migonei,* and other species, according to capture method and environment. Embu das Artes, July 2022 to December 2023.

Trap	Environment	Points	*Pintomyia fischeri*		*Migonemyia migonei*		Other species		Total
			N (%)	average	N (%)	average	N (%)	average	
Center for Disease Control	Domiciliary annex	**F**	20 (12.1)	1.11	14 (22.9)	0.78	4 (21.0)	0.22	38
	Veranda	**E**	114 (69.1)	9.55	37 (60.7)	3.08	6 (31.6)	0.50	157
		**G**	11 (6.7)	0.92	6 (9.8)	0.50	0 (0.0)	0.0	17
	Extradomicile	**A**, **B**	20 (12.1)	0.83	4 (6.6)	0.17	9 (47.4)	0.50	33
	Subtotal		165 (100)		61 (100)		19 (100)		245 (20.0)
Shannon	Peridomicile	**H**, **I**	840 (77.5)	35.0	42 (40.8)	1.75	16 (88.9)	0.7	898(100)
	Extradomicile	**C**, **D**	79 (7.3)	6.6	0 (0.0)	0.0	2 (11.1)	0.2	81
	Subtotal		919 (100)	25.5	42 (100)	1.17	18 (100)	0.50	979 (80.0)
Total			1084 (88.6)		103 (8.4)		37 (3.0)		1224(100)

A total of 245 specimens were collected using CDC light traps. The species composition followed the same pattern observed in the Shannon traps, with *Pi. fischeri* accounting for 67% of the specimens, followed by *Mg. migonei* (25%). Notably, the CDC trap located near the kennel captured the highest density, accounting for 64% of all specimens collected with this method, whereas traps placed on the veranda and in the peridomicile captured substantially fewer individuals.

The highest numbers of specimens were collected from late winter through summer, coinciding with periods of higher accumulated rainfall. However, no significant correlation was observed between rainfall and the abundance of *Pi. fischeri* (Spearman's ρ = 0.21, p = 0.39), whereas a significant positive correlation was found for *Mg. migonei* (Spearman's ρ = 0.56, p = 0.02). No significant correlations were observed for the remaining climatic variables.

Out of 790 dissected females, flagellates were observed in one *Evandromyia* (*Ev.*) *edwardsi* specimen collected in October 2022. The overall flagellate infection rate was 1/790 (0.13%), whereas the infection rate for *Ev. edwardsi* was 1/3 (33.3%). However, the flagellate species could not be identified.

Our findings are consistent with those of previous studies conducted in the RMSP^4^ ^,^ ^10^ ^-^ ^12^. The occurrence of these species appears to be associated with areas where urban environments interface with dense Atlantic Forest vegetation, as also reported by Moschin^11^ in Cantareira State Park. Likewise, all species identified in the present study have previously been recorded in forested areas of the municipality of São Paulo, including Cantareira and Jaraguá State Parks, where a total of 23 species have been reported^11^.

The presence of *Pi. fischeri* and *Mg. migonei* in collections at the kennel highlights the cynophilic behavior of these species. Of the 157 specimens collected at the kennel, 73% were *Pi. fischeri* (90 females and 24 males) and 24% were *Mg. migonei* (12 females and 26 males), representing 10.5% and 35.9%, respectively, of the total number of each species collected across all sites. *Pi. fischeri* was the most abundant species at the kennel, similar to that reported by Galvis-Ovallos et al.^9^, who observed a frequency of 95% *Pi. fischeri* and only one specimen of *Mg. migonei* at this site. However, analysis of the distribution by capture site showed that *Mg. migonei* occurred at a high frequency in the kennel, similar to the findings of Castelo et al.^11^. These differences may be related to site characteristics and temporal variation in the distribution of these species. Higher abundances were observed in Shannon traps than in CDC light traps, reflecting both the anthropophilic behavior of *Pi. fischeri* and *Mg. migonei* and the differences between the two collection methods (active versus passive), as previously reported for other species, such as *Lu. longipalpis* ^13^. Among the Shannon traps, greater attractiveness was observed for the black trap, corroborating the findings of Galati et al.^6^, who also reported higher attractiveness (90.1%) for this trap in Serra da Bodoquena, Mato Grosso do Sul. 

Regarding temporal distribution, higher densities of *Pi. fischeri* and *Mg. migonei* were observed from late winter through summer, suggesting that increased temperature and precipitation favor the development of these species^10^.

The predominance of *Pi. fischeri* in this study reinforces previous evidence supporting its role as a vector of the VL agent in the region, as previously reported for the RMSP^4^ ^,^ ^8^. Another noteworthy finding is the high frequency of *Mg. migonei* in the kennel compared to that in the other capture sites. Although this species represented only 8.4% of the total specimens collected, 35% were captured at the kennel suggesting a marked cynophilic behavior. It also supports previous evidence of its role as a vector of the VL agent based on studies conducted in Pernambuco, including the detection of natural infection with *Le. Infantum* ^14^ and demonstrated vector competence^9^. Our findings support the hypothesis of an alternative local transmission cycle in the absence of *Lu. longipalpis*, reinforcing the need for further studies on the role of secondary vectors in the region.

Detecting parasite circulation in vector populations is an important indicator of local parasite transmission and provides evidence for the incrimination of species present in a given focus^5^. In the present study, although flagellates were detected in one *Ev. edwardsi* female, the parasite could not be identified. Nevertheless, this finding is noteworthy considering that only three females were collected. Previous studies conducted in the municipality of Cotia also reported low densities of this species and detected one female naturally infected with *Le. braziliensis* ^13^. These findings highlight the need for further studies on the role of this species in the transmission of *Leishmania*, particularly using alternative collection methods.

Some limitations of this study should be acknowledged. Collections were conducted at a single household, which may not fully represent the diversity and abundance of sand flies throughout the municipality. In addition, changes in trap locations during the study period may have influenced temporal comparisons and species diversity. Furthermore, although flagellates were detected in one *Ev. edwardsi* female, parasite identification was unsuccessful, limiting the strength of the evidence obtained.

In summary, this study suggests that a diverse sand fly community may sustain leishmaniasis transmission in the area with *Pi. fischeri* as the predominant species and the primary putative vector in the region. The detection of flagellates in *Ev. edwardsi*, together with the marked attraction of *Mg. migonei* to canine environments, suggests a complex transmission cycle involving multiple sand fly species acting as primary and secondary vectors.

The absence of the primary vector, *Lu. longipalpis* indicates that the epidemiology of leishmaniasis in this locality differs from that of other endemic regions, underscoring the importance of local studies to guide control strategies. The strong association of *Pi. fischeri* and *Mg. migonei* with the kennel highlight the role of dogs as potential sources of infection and the need to strengthen preventive measures targeting these reservoirs, thus emphasizing the importance of integrating entomological surveillance with prevention and control activities in the municipality.

## Data Availability

Research data is available in the body of the document. (Figure, Tables and from the last paragraph at page four at page five).
